# Association between thyroid hormones and cognitive functioning in euthyroid elderly adults: a cross-sectional preliminary study from the NHANES 2011–2012 survey

**DOI:** 10.3389/fendo.2024.1476086

**Published:** 2024-11-08

**Authors:** Bingbing Xv, Peiyun Wu, Ping Li, Wanling Chen, Xiangzhi Rao, Boqi Lu, Cheng Lin, Zhizhuo Wang

**Affiliations:** Department of Rehabilitation Medicine, School of Health, Fujian Medical University, Fuzhou, China

**Keywords:** thyroid hormones, cognitive functioning, elderly euthyroid adults, CERAD W-L, animal fluency test, DSST

## Abstract

**Objective:**

Changes in serum thyroid hormone levels may affect cognitive functioning in euthyroid individuals. This study used representative data from the National Health and Nutrition Examination Survey (NHANES) to comprehensively examine the association of thyroid hormones with different tests of cognitive functioning among US elderly people aged ≥60 years.

**Methods:**

This study was a cross-sectional preliminary study with a total of 734 participants from the NHANES 2011-2012 survey. Thyroid function was measured using competitive binding immune-enzymatic assays, while cognitive functioning was measured using a series of assessments, including the Consortium to Establish a Registry for Alzheimer’s Disease-Word Learning (CERAD W-L), Animal Fluency Test, and Digit Symbol Substitution Test (DSST). Weighted multiple linear regression models and binary logistic regression analyses were used to examine the association between thyroid hormone levels and cognitive functioning. All statistical analyses were performed using SPSS version 20.0, and R software.

**Results:**

Weighted multivariable linear regression showed that FT3 was negatively associated with the Animal Fluency Test and DSST (β=-0.113, 95% CI: -3.279, -0.803, P=0.001; β=-0.062, 95% CI: -6.565, -0.470, P=0.024, respectively) after adjustment for potential covariates. subgroup analysis stratified by sex revealed a negative association between FT3 levels and the Animal Fluency Test in men (β=-0.163, 95% CI: -4.643, -1.153, P=0.001). For female participants, FT3 was negatively associated with not only the Animal Fluency Test but also DSST (β=-0.099, 95% CI: -3.543, -0.093, P=0.039; β=-0.093, 95% CI: -10.288, -1.326, P=0.011). Binary logistic regression showed that the significantly increased adjusted odds ratios (aORs) (95% CI) between the risk of impaired cognitive functioning and FT3 across Q3 and Q4 compared with Q1 were 2.025 (1.092, 3.753) and 2.365 (1.261, 4.433), respectively, for DSST in overall participants. Furthermore, there were significant differences between participants with and without impaired cognitive functioning for serum FT3 levels in overall participants based on DSST score (P=0.020).

**Conclusions:**

There was a significant inverse relationship between FT3 levels within the normal range and cognitive functioning after adjusting for potential covariates. Future longitudinal cohort studies should be conducted to determine the causal relationship between thyroid hormone levels and cognitive functioning.

## 1 Introduction

Cognitive impairment refers to poor behavioral performance in one or more cognitive domains that may not interfere with individual’s daily tasks absolutely (1). Dementia or major neurocognitive disorder (MNCD) is marked by gradually occurring amnesia and a loss of cognitive abilities, which contributes to significant functional impairment (2). On a global scale, the prevalence of dementia appears to be twofold every 5 years for adults aged between 50 and 80 years. In the United States, the prevalence of dementia in individuals aged ≥ 68 years is 15% and is expected to increase as life expectancy increases (3). Therefore, identifying and managing cognitive impairment in older adults is of great importance.

Thyroid hormones (THs) play a critical role in maintaining the differentiation, growth, and metabolism of both animal and human organ systems. Evidence has shown that both subclinical hypothyroidism and hyperthyroidism are closely related to cognitive impairment (4, 5). Previous studies have confirmed that changes in serum THs may affect cognitive functioning in euthyroid populations (6, 7). Although several studies have reported associations between THs in the reference range and cognitive functioning and mood in healthy elderly people, their findings are inconsistent. For example, Grigorova and Sherwin found that higher levels of free triiodothyronine (FT3) and thyroglobulin antibodies (TgAb) within the normal range may negatively exert influence on executive functions (8). In contrast, an inverse linear association between serum FT3 within the normal range and risk of Alzheimer’s disease (AD) was reported in a prospective cohort study (9). The prospective, population-based Korean Longitudinal Study on Health and Aging (KLoSHA) revealed that lower serum thyroid-stimulating hormone (TSH) levels within the reference range were associated with the risk of cognitive dysfunction, including mild cognitive impairment (MCI) and dementia in euthyroid elderly people (10). Conversely, other studies found that higher serum TSH within the normal range was associated with poorer cognitive performance (11, 12). However, van Vliet et al. failed to find a consistent association between thyroid dysfunction and global cognitive function, executive function, memory, and risk of dementia in elderly participants (13).

In total, given that the results of previous studies were controversial, and few studies have focused on the US population, the current study used representative national data from the National Health and Nutrition Examination Survey (NHANES) to comprehensively examine the association of thyroid hormones with different tests of cognitive functioning among US elderly people aged ≥60 years.

## 2 Materials and methods

### 2.1 Study population

**Figure 1:**
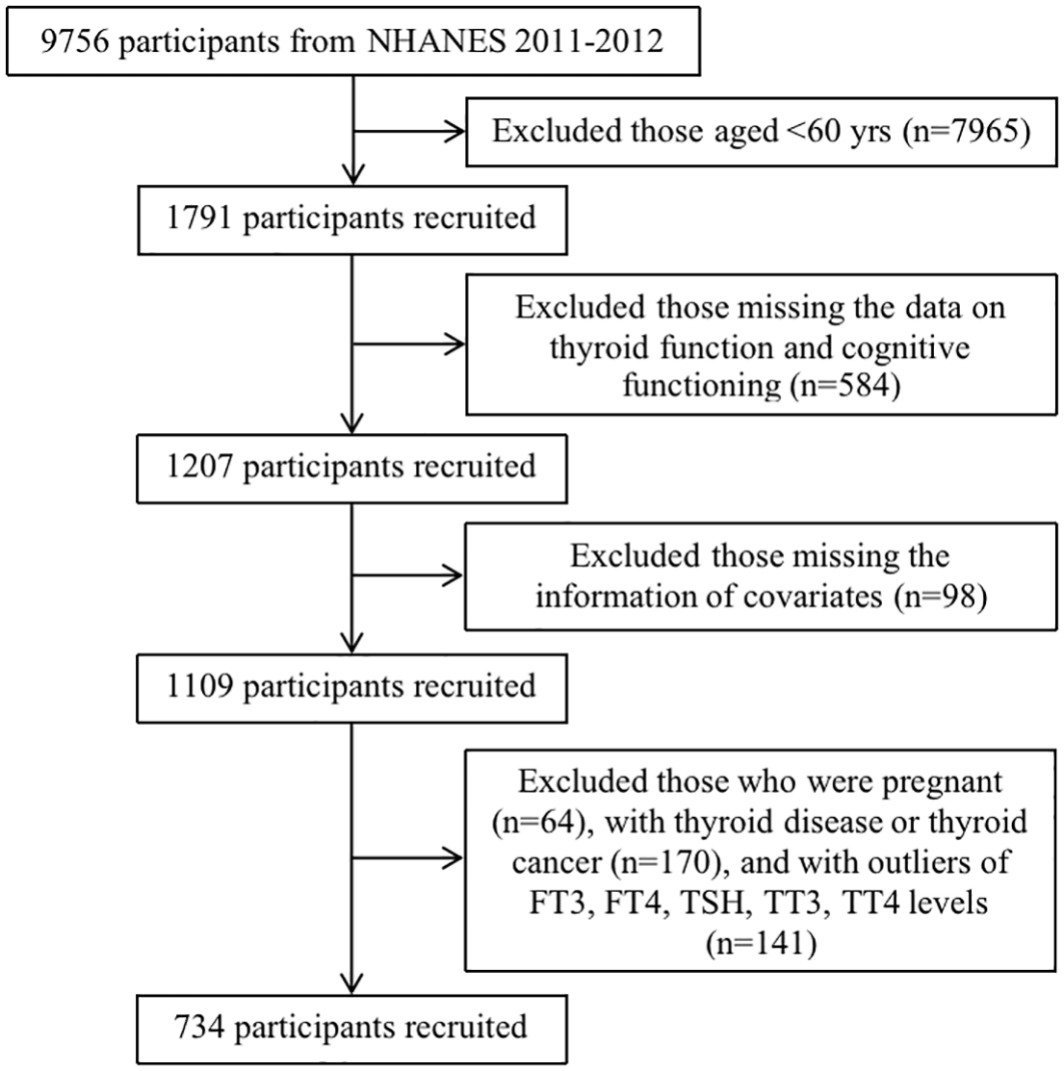
The flowchart of participants’ inclusion and exclusion process.

The National Health and Nutrition Examination Survey (NHANES) is a nationally representative, two-year cycle cross-sectional survey that employs a complex multistage probability sampling design among the civilian, non-institutionalized U.S. population. The NHANES was approved by the National Center for Health Statistics Research Ethics Review Board. The data were retrieved from the NHANES 2011-2012 survey cycle, in which intact files on thyroid function and cognitive functioning information were embodied. A total of 9,756 participants in the NHANES 2011-2012 were included. We excluded participants aged <60 years (n=7,965), those without data related to the independent variables of interest (i.e., thyroid function and cognitive functioning) (n=584), and those with missing covariate information, including age, sex, race/ethnicity, educational level, marital status, smoking status, drinking status, hypertension, BMI, UIC, creatinine, copper, selenium, and zinc (n=98). Women who were pregnant (n=64), those with thyroid disease or thyroid cancer (n=170), and those with outliers in FT3, FT4, TSH, TT3, and TT4 levels (n=141) were excluded. Ultimately, 734 participants were included in the final analysis (**Figure 1**).

### 2.2 Thyroid hormones

Serum FT3, FT4, TSH, TT3, and TT4 levels were obtained from the NHANES laboratory sections. A competitive binding immunoenzymatic assay was used to measure FT3, FT4, TT3, and TT4. The measurements were performed using a third-generation, two-site immune-enzymatic (“sandwich”) assay. A detailed description of the laboratory methodology is provided at www.cdc.gov/Nchs/Nhanes/2011-2012/THYROD_G.htm. The reference ranges of FT3, FT4, TSH, TT3 and TT4 were 2.5~3.9 pg/ml, 0.6~1.6 ng/dl, 0.34~5.6 μIU/ml, 80~220 ng/dl and 5.0~12.0 ug/dl, respectively (14).

### 2.3 Cognitive functioning

Cognitive functioning was measured using a series of assessments in the NHANES 2011-2012, including: 1) word learning and recall modules from the Consortium to Establish a Registry for Alzheimer’s Disease (CERAD), 2) the Animal Fluency Test, and 3) the Digit Symbol Substitution Test (DSST).

The CERAD Word Learning subtest (CERAD W-L) evaluates immediate and delayed learning ability for new verbal information (15, 16). The CERAD W-L comprises three consecutive learning trials and delayed recall. Regarding the learning trials, participants were instructed to read 10 unrelated words aloud once, followed by recalling as many words as possible immediately after their presentation. Delayed word recall occurred approximately 8-10 minutes after the inception of the word learning trials. The maximum possible score for each trial was 10 and the maximum possible score for the CERAD W-L test was 40.

The Animal Fluency Test examines verbal category fluency, a component of executive function (17). Participants were asked to name as many animals as possible in one minute. A point was assigned to each animal. Scores have been shown to discriminate between individuals with normal cognitive functioning and those with mild cognitive impairment and/or more severe cognitive impairment, namely Alzheimer’s disease (18–20).

The Digit Symbol Substitution Test (DSST) is a performance module from the Wechsler Adult Intelligence Scale (WAIS III), which relies on processing speed, visual scanning, sustained attention, and working memory (21). The test is conducted using a paper form with a key at the top containing nine numbers paired with recognizable symbols. Participants were given two minutes to copy the corresponding symbols in the 133 boxes that neighbor the numbers. The score is based on the total number of correct matches.

Currently, no consensus has been reached on the standardized cutoff points for the CERAD W-L, Animal Fluency Test and DSST for determining impaired cognitive functioning. As such, we used the lowest quartile of the score (i.e., 25th percentile) as the cutoff point according to previous published literature (22). Moreover, the cutoff point was set based on different age groups (60~69 yrs, 70~79 yrs, and ≥80 yrs) in view of the significant effect of age on cognitive functioning (23). Specifically, the cutoff values of CERAD W-L were 22, 19 and 14 for the three age groups, respectively. Similarly, for the three age groups, the cutoff values of the Animal Fluency Test were 14, 12, and 11, and the cutoff values of the DSST were 35, 30, and 26, respectively. For each cognitive functioning test, participants were divided into two groups: the impaired cognitive functioning group, in which participants scored lower than the corresponding cutoff values, and the normal cognitive functioning group, to which the rest of the participants were assigned.

### 2.4 Covariates

The covariates considered in the study were as follows: sex (male, female), race/ethnicity (Mexican American, other Hispanics, non-Hispanics, other races), educational level (below high school, high school, and above high school), marital status (married/living with partner, widowed/divorced/separated/never married), drinking status (yes, no), smoking status (never, former, current), hypertension (yes, no), urine iodine concentration (UIC, categorized into <100ug/L as iodine deficient, 100~299ug/L as normal, and ≥300ug/L as excessive iodine intake), body mass index (BMI, grouped into <25kg/m^2^ as normal, 25~29.9 kg/m^2^ as overweight, and ≥30kg/m^2^ as obese), creatinine (mg/dL), copper (µg/dL), selenium (ug/dL), and zinc (µg/dL). The “Smoking–Cigarette Use” questionnaire is used to determine the smoking status of participants (24). Hypertension is defined as blood pressure ≥140/90 mmHg, or self-reported previous diagnosis of hypertension, or self-reported use of antihypertensive medication (25). BMI is defined as weight in kilograms divided by the square of height in meters, and the classification refers to prior published literature (26). UIC is divided into three different groups in accordance with iodine concentration, which could impact thyroid function (27).

### 2.5 Statistical analysis

The survey sampling weights were considered because of the complex multistage probability sampling design. For the basic characteristics of the participants, continuous variables were reported as the weighted mean ± standard deviation (SD), while categorical variables were expressed as counts (weighted percentages). If the variable conformed to a normal distribution, a t-test was used to compare differences between the impaired and normal cognitive functioning groups. Otherwise, the Mann–Whitney U test was used. For categorical variables, the chi-square test was used. Weighted multivariable linear regression models were applied to test the association between thyroid function, including two indices (FT3, FT4, TSH, TT3, and TT4), and cognitive functioning (CERAD W-L Subtest, Animal Fluency Test, and DSST). Model 1 was crudely adjusted (unadjusted); Model 2 was adjusted for age, sex, race/ethnicity, educational level, and marital status; and Model 3 was additionally adjusted for smoking status, drinking status, hypertension, BMI, UIC, creatinine, copper, selenium, and zinc. Subgroup analyses stratified by sex were performed with all covariates except those adjusted. Furthermore, we divided the FT3, FT4, TSH, TT3, and TT4 levels into four categories based on the quartiles of each variable. Binary logistic regression analyses were conducted to examine the association between thyroid function and cognitive functioning after adjusting for all aforementioned confounding factors. The missing data on investigated variables (i.e., thyroid function and cognitive functioning) and covariates were examined to see whether they were distributed equally across socio-demographic characteristics, and no statistically significant differences were found (all P>0.05). As such, it is acceptable to continue the analysis without further imputation of missing values. All statistical analyses were performed using SPSS (version 20.0; SPSS Inc., Chicago, IL, USA) and R software (version 4.1.0; R Foundation for Statistical Computing, Vienna, Austria). Differences were considered statistically significant at P<0.05.

## 3 Results

Of the 734 participants, the majority were men (55.04%), non-Hispanic White or Black (78.47%), with an educational level above high school (48.91%), and married or living with partner (58.86%). The participants were primarily current smokers (47.55%), self-reported alcohol consumers (69.35%), and those diagnosed with hypertension (69.75%). For most participants, BMI (36.24%) and UIC (48.64%) were high, in the ranges of ≥30 kg/m^2^ and 100~299ug/L, respectively. As shown in **Table 1**, there were significant differences (P<0.05) between participants with normal cognitive functioning and those with impaired cognitive functioning in terms of sex, race/ethnicity, educational level, marital status, smoking status, Animal Fluency Test results and hypertension. However, no statistically significant differences were found in age, drinking status, BMI, or UIC, creatinine, copper, selenium, or zinc levels between participants with and without impaired cognitive performance (all P>0.05).

**Table 1:** The basic characteristics of participants.

Variables	CERAD W-L Subtest			Animal Fluency Test			DSST		
	Normal cognitive functioning (n=570)	Impaired cognitive functioning (n=164)	P value	Normal cognitive functioning (n=566)	Impaired cognitive functioning (n=168)	P value	Normal cognitive functioning (n=564)	Impaired cognitive functioning (n=170)	P value
Age (yrs), n (%)			0.959			0.475			0.925
60~69	330 (60.46)	95 (49.82)		322 (59.92)	103 (51.44)		326 (58.75)	99 (57.04)	
70~79	161 (26.22)	45 (34.37)		165 (27.53)	41 (28.96)		160 (27.28)	46 (29.93)	
≥80	79 (13.32)	24 (15.81)		79 (12.55)	24 (19.60)		78 (13.97)	25 (13.03)	
Sex, n (%)			**<0.001**			0.787			0.191
Male	294 (53.10)	110 (70.96)		310 (56.45)	94 (56.86)		303 (55.17)	101 (62.28)	
Female	276 (46.90)	54 (29.04)		256 (43.55)	74 (43.14)		261 (44.83)	69 (37.72)	
Race/Ethnicity, n (%)			**0.004**			0.263			**<0.001**
Mexican American	37 (5.89)	19 (9.08)		42 (5.84)	14 (9.54)		36 (5.28)	20 (11.70)	
Other Hispanic	58 (9.44)	30 (14.04)		61 (9.61)	27 (13.64)		40 (6.80)	48 (25.37)	
Non-Hispanic	463 (82.61)	113 (76.33)		453 (82.66)	123 (75.58)		476 (85.85)	100 (62.43)	
Other Race	12 (2.07)	2 (0.55)		10 (1.89)	4 (1.25)		12 (2.07)	2 (0.50)	
Educational level, n (%)			**<0.001**			**<0.001**			**<0.001**
Below high school	137 (20.68)	79 (45.04)		143 (21.41)	73 (43.70)		105 (16.81)	111 (61.83)	
High school	121 (20.20)	38 (21.00)		114 (19.54)	45 (24.20)		129 (19.97)	30 (22.02)	
Above high school	312 (59.12)	47 (33.96)		309 (59.05)	50 (32.10)		330 (63.22)	29 (16.15)	
Marital status, n (%)			0.320			0.489			**0.020**
Married/Living with partner	341 (62.21)	91 (60.15)		337 (62.83)	95 (57.10)		345 (64.95)	87 (48.42)	
Widowed/Divorced/Separated/Never married	229 (37.79)	73 (39.85)		229 (37.17)	73 (42.90)		219 (35.05)	83 (51.58)	
Smoking status, n (%)			**0.039**			0.872			0.133
Never	78 (13.55)	35 (17.32)		85 (14.25)	28 (14.35)		79 (13.97)	34 (15.55)	
Former	211 (35.34)	61 (43.57)		211 (37.41)	61 (34.61)		209 (36.59)	63 (38.31)	
Current	281 (51.11)	68 (39.11)		270 (48.34)	79 (51.04)		276 (49.44)	73 (46.14)	
Drinking status, n (%)			0.807			0.153			0.191
Yes	394 (72.36)	115 (73.97)		400 (74.89)	109 (62.28)		398 (74.06)	111 (66.72)	
No	176 (27.64)	49 (26.03)		166 (25.11)	59 (37.72)		166 (25.94)	59 (33.28)	
Hypertension, n (%)			**0.015**			0.092			**0.002**
Yes	385 (66.30)	127 (78.17)		386 (67.35)	126 (74.27)		377 (65.45)	135 (81.88)	
No	185 (33.70)	37 (21.83)		180 (32.65)	42 (25.73)		187 (34.55)	35 (18.12)	
BMI, n (%)			0.244			0.684			0.909
<25kg/m^2^ (Normal)	160 (27.32)	56 (30.80)		164 (27.06)	52 (32.31)		165 (28.44)	51 (26.08)	
25-29.9kg/m^2^ (Overweight)	203 (37.54)	49 (30.63)		199 (37.82)	53 (28.72)		196 (37.27)	56 (31.69)	
≥30kg/m^2^ (Obese)	207 (35.14)	59 (38.57)		203 (35.12)	63 (38.97)		203 (34.29)	63 (42.23)	
UIC, n (%)			0.092			0.170			0.337
<100μg/L (Iodine deficient)	201 (33.89)	44 (25.14)		182 (30.87)	63 (38.49)		190 (31.55)	55 (35.07)	
100~299μg/L (Normal)	266 (49.79)	91 (57.47)		286 (53.69)	71 (39.90)		279 (52.51)	78 (45.90)	
≥300μg/L (Excessive iodine intake)	103 (16.32)	29 (17.39)		98 (15.44)	34 (21.61)		95 (15.94)	37 (19.03)	
Creatinine (mg/dL), mean ± SD	105.10 ± 68.37	116.80 ± 71.47	0.410	105.57 ± 67.00	115.64 ± 77.77	0.234	107.53 ± 70.13	106.52 ± 64.65	0.489
Copper (μg/dL), mean ± SD	113.57 ± 22.64	116.39 ± 24.72	0.980	114.40 ± 22.44	112.72 ± 25.81	0.674	113.76 ± 22.66	115.59 ± 24.72	0.967
Selenium (μg/dL), mean ± SD	130.26 ± 17.94	128.56 ± 19.52	0.750	130.36 ± 17.83	127.94 ± 20.06	0.628	130.51 ± 17.80	127.48 ± 19.95	0.378
Zink (μg/dL), mean ± SD	82.22 ± 13.85	81.46 ± 15.68	0.366	82.51 ± 14.57	80.04 ± 12.28	0.311	82.27 ± 14.12	81.24 ± 14.64	0.506

Bolded values were statistically significant (P<0.05).

CERAD, Consortium to Establish a Registry for Alzheimer’s Disease; CERAD W-L, CERAD Word Learning; DSST, Digit Symbol Substitution test; BMI, Body mass index; UIC, Urine iodine concentration; FT3, Free triiodothyronine; FT4, Free thyroxine; TT3, Total triiodothyronine; TT4, Total thyroxine; TSH, Thyroid-stimulating hormone.

As shown in **Table 2**, weighted multivariable linear regression was employed to evaluate the association between thyroid function and cognitive functioning in adults aged ≥60 years. In the crude model, FT3 and TT3 were negatively associated with the Animal Fluency Test (β=-0.152, 95% CI: -11.252, -3.163, P<0.001; β=-0.099, 95% CI: -0.051, -0.005, P=0.015, respectively). In addition, FT3, TT3 and TT4 were negatively associated with DSST (β=-0.128, 95% CI: -11.252, -3.163, P<0.001; β=-0.087, 95% CI: -0.146, -0.009, P=0.027; β=-0.111, 95% CI: -2.414, -0.485, P=0.003, respectively). After adjusting for age, sex, race/ethnicity, educational level, and marital status in Model 2, only FT3 remained significantly negatively associated with the Animal Fluency Test and DSST (β=-0.130, 95% CI: -3.576, -1.122, P<0.001; β=-0.070, 95% CI: -7.034, -0.889, P=0.012, respectively). Furthermore, when employing a fully adjusted model (Model 3), we found that FT3 remained significant in the Animal Fluency Test and DSST (β=-0.113, 95% CI: -3.279, -0.803, P=0.001; β=-0.062, 95% CI: -6.565, -0.470, P=0.024, respectively). Interestingly, no statistically significant association was found between thyroid function (FT3, FT4, TSH, TT3, and TT4) and the CERAD W-L Subtest (all P>0.05).

**Table 2:** Weighted multivariable linear regression analysis of the association between thyroid hormones and cognitive functioning.

Thyroid hormones	CERAD W-L Subtest			Animal Fluency Test			DSST		
Model 1									
	Standardized β	95% CI	P value	Standardized β	95% CI	P value	Standardized β	95% CI	P value
FT3	0.008	(-1.287, 1.627)	0.819	-0.152	(-4.096, -1.401)	**<0.001**	-0.128	(-11.252, -3.163)	**<0.001**
FT4	-0.030	(-5.100, 2.108)	0.415	-0.047	(-5.476, -1.257)	0.219	-0.035	(-14.983, 5.184)	0.340
TT3	0.010	(-0.021, 0.028)	0.802	-0.099	(-0.051, -0.005)	**0.015**	-0.087	(-0.146, -0.009)	**0.027**
TT4	-0.056	(-0.607, -0.086)	0.140	-0.066	(-0.600, 0.047)	0.093	-0.111	(-2.414, -0.485)	**0.003**
TSH	-0.019	(-0.642, 0.386)	0.625	-0.0006	(-0.484, 0.477)	0.988	-0.0005	(-1.448, 1.429)	0.990
Model 2									
	Standardized β	95% CI	P value	Standardized β	95% CI	P value	Standardized β	95% CI	P value
FT3	0.034	(-0.567, 1.934)	0.284	-0.130	(-3.576, -1.122)	**<0.001**	-0.070	(-7.034, -0.889)	**0.012**
FT4	-0.005	(-3.306, 2.802)	0.871	-0.039	(-4.751, 1.292)	0.261	-0.004	(-8.076, 6.988)	0.887
TT3	0.034	(-0.010, 0.032)	0.315	-0.063	(-0.039, 0.003)	0.089	-0.024	(-0.073, 0.030)	0.412
TT4	-0.012	(-0.352, 0.238)	0.703	-0.020	(-0.376, 0.208)	0.571	-0.049	(-1.370, 0.082)	0.082
TSH	0.014	(-0.347, 0.531)	0.681	0.010	(-0.372, 0.496)	0.780	0.003	(-1.018, 1.146)	0.908
Model 3									
	Standardized β	95% CI	P value	Standardized β	95% CI	P value	Standardized β	95% CI	P value
FT3	0.034	(-0.585, 1.963)	0.289	-0.113	(-3.279, -0.803)	**0.001**	-0.062	(-6.565, -0.470)	**0.024**
FT4	-0.011	(-3.704, 2.578)	0.725	-0.051	(-5.347, 0.790)	0.145	-0.001	(-7.727, 7.345)	0.960
TT3	0.035	(-0.010, 0.032)	0.311	-0.057	(-0.037, 0.004)	0.124	-0.011	(-0.061, 0.042)	0.707
TT4	-0.005	(-0.329, 0.282)	0.879	-0.018	(-0.375, 0.223)	0.617	-0.034	(-1.171, 0.295)	0.241
TSH	0.019	(-0.322, 0.581)	0.573	-0.010	(-0.498, 0.385)	0.802	-0.011	(-1.289, 0.876)	0.708

Model 1: unadjusted; Model 2: age, sex, race/ethnicity, educational level, marital status were adjusted; Model 3: age, sex, race/ethnicity, educational level, marital status, smoking status, drinking status, hypertension, BMI, UIC, Creatinine, Copper, Selenium, Zinc were adjusted; Bolded values were statistically significant (P<0.05).

Subgroup analysis stratified by sex was conducted to further examine the relationship between thyroid and cognitive functioning. After adjusting for all confounding factors except sex, subgroup analysis by sex (**Table 3**) revealed a negative association between FT3 and the Animal Fluency Test for men (β=-0.163, 95% CI: -4.643, -1.153, P=0.001). For female participants, FT3 was negatively associated with not only the Animal Fluency Test but also the DSST (β=-0.099, 95% CI: -3.543, -0.093, P=0.039; β=-0.093, 95% CI: -10.288, -1.326, P=0.011, respectively).

**Table 3:** Subgroup analysis stratified by sex of the association between thyroid hormones and cognitive functioning.

Thyroid hormones	CERAD W-L Subtest			Animal Fluency Test			DSST		
Male (Model 3)									
	Standardized β	95% CI	P value	Standardized β	95% CI	P value	Standardized β	95% CI	P value
FT3	0.084	(-0.218, 3.348)	0.085	-0.163	(-4.643, -1.153)	**0.001**	-0.050	(-6.902, 1.795)	0.249
FT4	-0.050	(-6.335, 1.634)	0.247	-0.019	(-4.810, 3.079)	0.666	0.007	(-8.823, 10.605)	0.857
TT3	0.026	(-0.020, 0.034)	0.611	-0.070	(-0.045, 0.009)	0.181	-0.003	(-0.068, 0.064)	0.948
TT4	-0.037	(-0.563, 0.246)	0.441	0.005	(-0.378, 0.422)	0.914	-0.015	(-1.154, 0.815)	0.735
TSH	0.030	(-0.429, 0.795)	0.557	-0.039	(-0.831, 0.379)	0.463	-0.048	(-2.274, 0.702)	0.300
Female (Model 3)									
	Standardized β	95% CI	P value	Standardized β	95% CI	P value	Standardized β	95% CI	P value
FT3	-0.033	(-2.621, 1.213)	0.470	-0.099	(-3.543, -0.093)	**0.039**	-0.093	(-10.288, -1.326)	**0.011**
FT4	0.052	(-2.567, 7.914)	0.316	-0.097	(-9.008, 0.447)	0.076	-0.016	(-14.769, 9.997)	0.705
TT3	0.043	(-0.020, 0.052)	0.371	-0.068	(-0.055, 0.010)	0.176	-0.029	(-0.117, 0.052)	0.451
TT4	0.059	(-0.183, 0.764)	0.229	-0.060	(-0.685, 0.172)	0.240	-0.041	(-1.713, 0.524)	0.296
TSH	-0.003	(-0.712, 0.665)	0.947	0.010	(-0.561, 0.686)	0.844	0.030	(-1.003, 2.244)	0.453

The results of the subgroup analysis were adjusted for age, race/ethnicity, educational level, marital status, smoking status, drinking status, hypertension, BMI, UIC, Creatinine, Copper, Selenium, Zinc (sex was not adjusted in this model); bold values are statistically significant (P<0.05).

**Figure 2:**
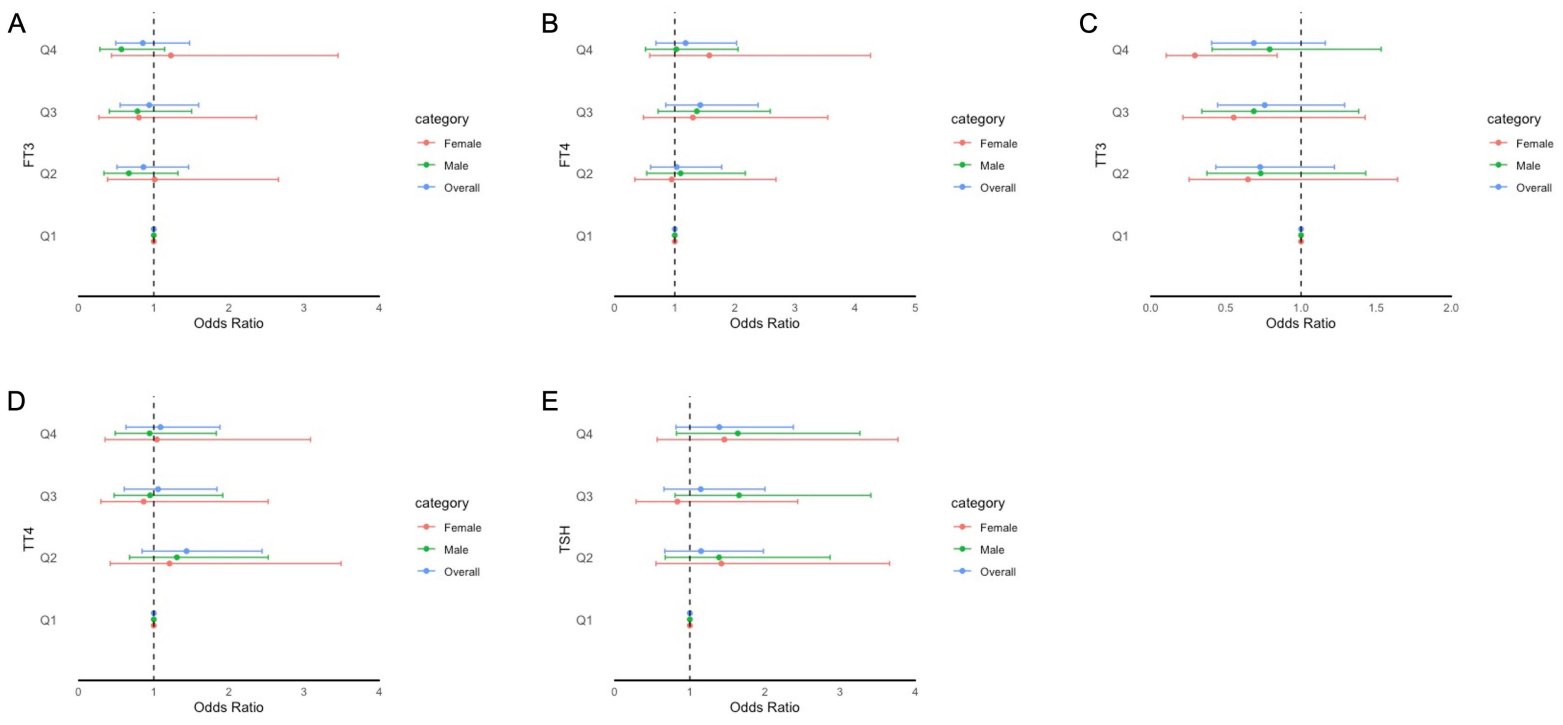
Forest plot of stratified analysis of the association between thyroid hormones and cognitive functioning based on CERAD W-L Subtest. **(A)** for FT3; **(B)** for FT4; **(C)** for TT3; **(D)** for TT4; **(E)** for TSH.

**Figure 3:**
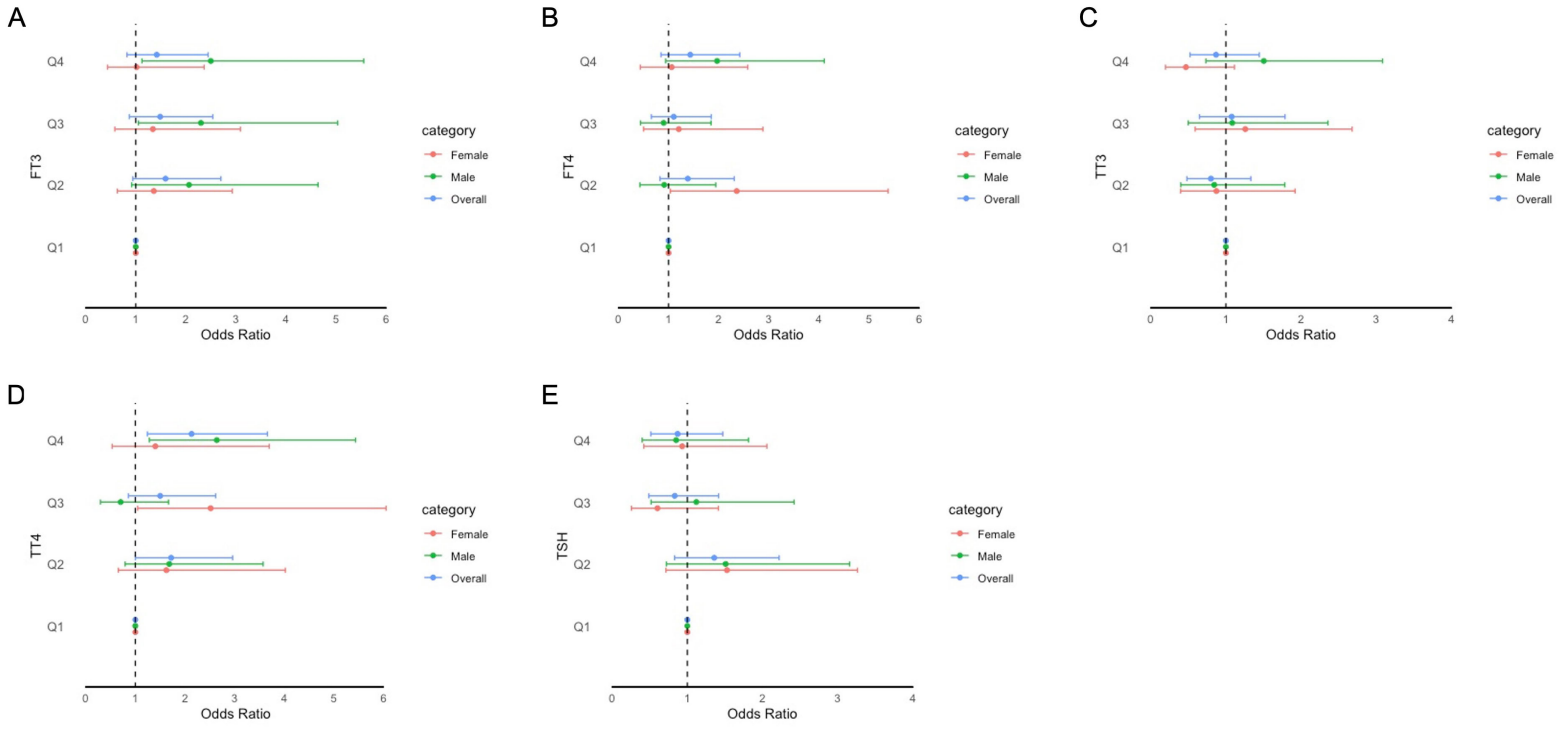
Forest plot of stratified analysis of the association between thyroid hormones and cognitive functioning based on Animal Fluency Test. **(A)** for FT3; **(B)** for FT4; **(C)** for TT3; **(D)** for TT4; **(E)** for TSH.

**Figure 4:**
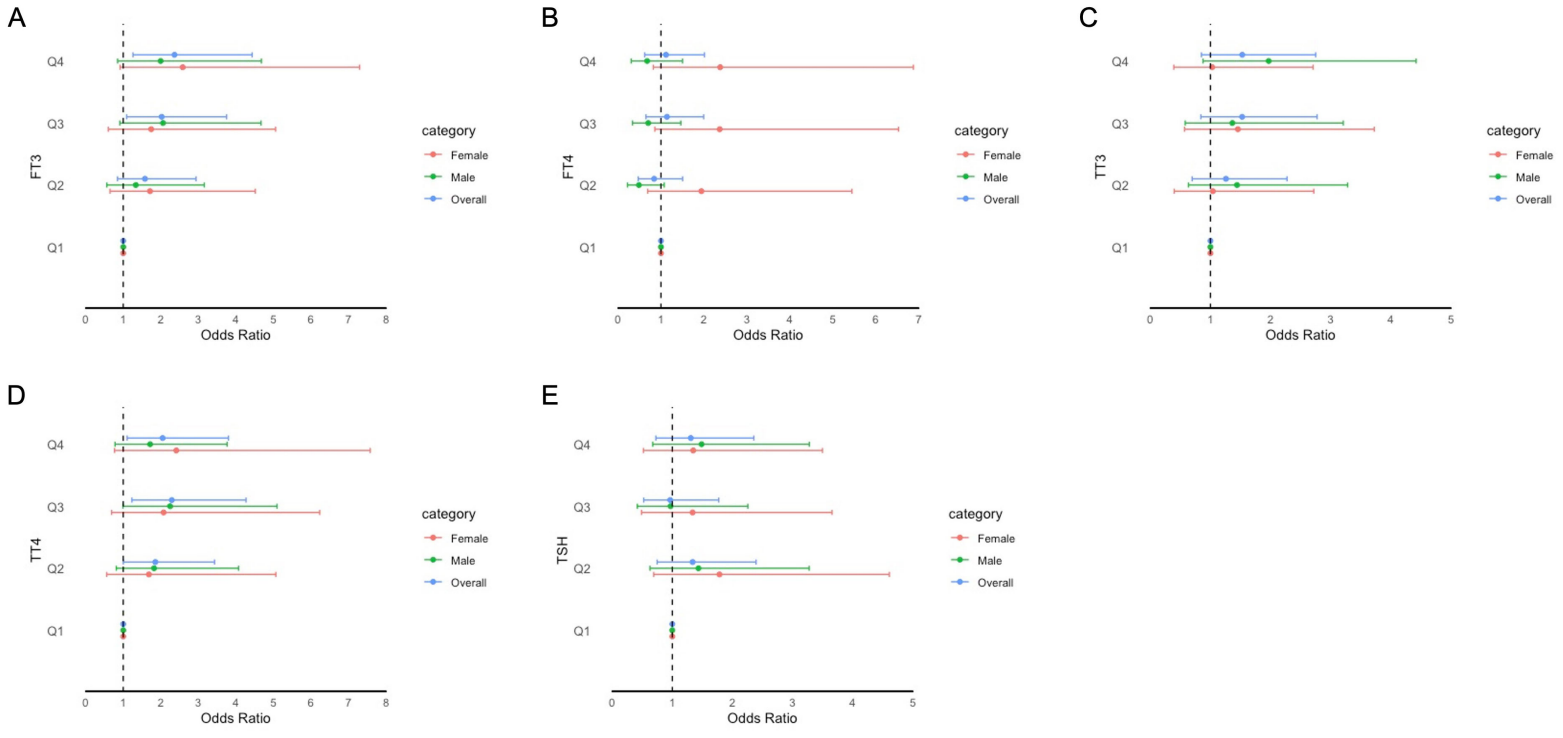
Forest plot of stratified analysis of the association between thyroid hormones and cognitive functioning based on DSST. **(A)** for FT3; **(B)** for FT4; **(C)** for TT3; **(D)** for TT4; **(E)** for TSH.

The adjusted odds ratios (aORs) for the risk of impaired cognitive functioning according to the FT3, FT4, TSH, TT3, and TT4 quartiles are presented in **Figures 2**–**4**. For the CERAD W-L Subtest, compared to the lowest quartile (Q1) of TT3 level, the aOR (95% CI) of Q4 was 0.294 (0.103, 0.841) in women. For the Animal Fluency Test, compared to Q1 of the TT4 level, the aOR (95% CI) of Q4 was 2.133 (1.243, 3.660) in all participants, and the aORs of Q3 and Q4 were 2.517 (1.047, 6.050) and 2.640 (1.282, 5.435) in female and male participants, respectively. In addition, compared to Q1 of FT3 level, aORs (95% CI) of Q3 and Q4 were 2.302 (1.053, 5.033) and 2.499 (1.125, 5.554) in men. In women, the aORs of Q2 (for FT4 level) and Q3 (for TT4 level) were 2.360 (1.035, 5.382) and 2.517 (1.047, 6.050), respectively, in comparison to Q1 of FT4 and TT4 levels. Regarding DSST in overall participants, the significantly increased aORs (95% CI) between the risk of impaired cognitive functioning and FT3 across Q3 and Q4 compared with Q1were 2.025 (1.092, 3.753) and 2.365 (1.261, 4.433), respectively. Furthermore, significant association between the risk of impaired cognitive functioning and TT4 were found between Q2 and Q4 compared to Q1.

**Figure 5:**
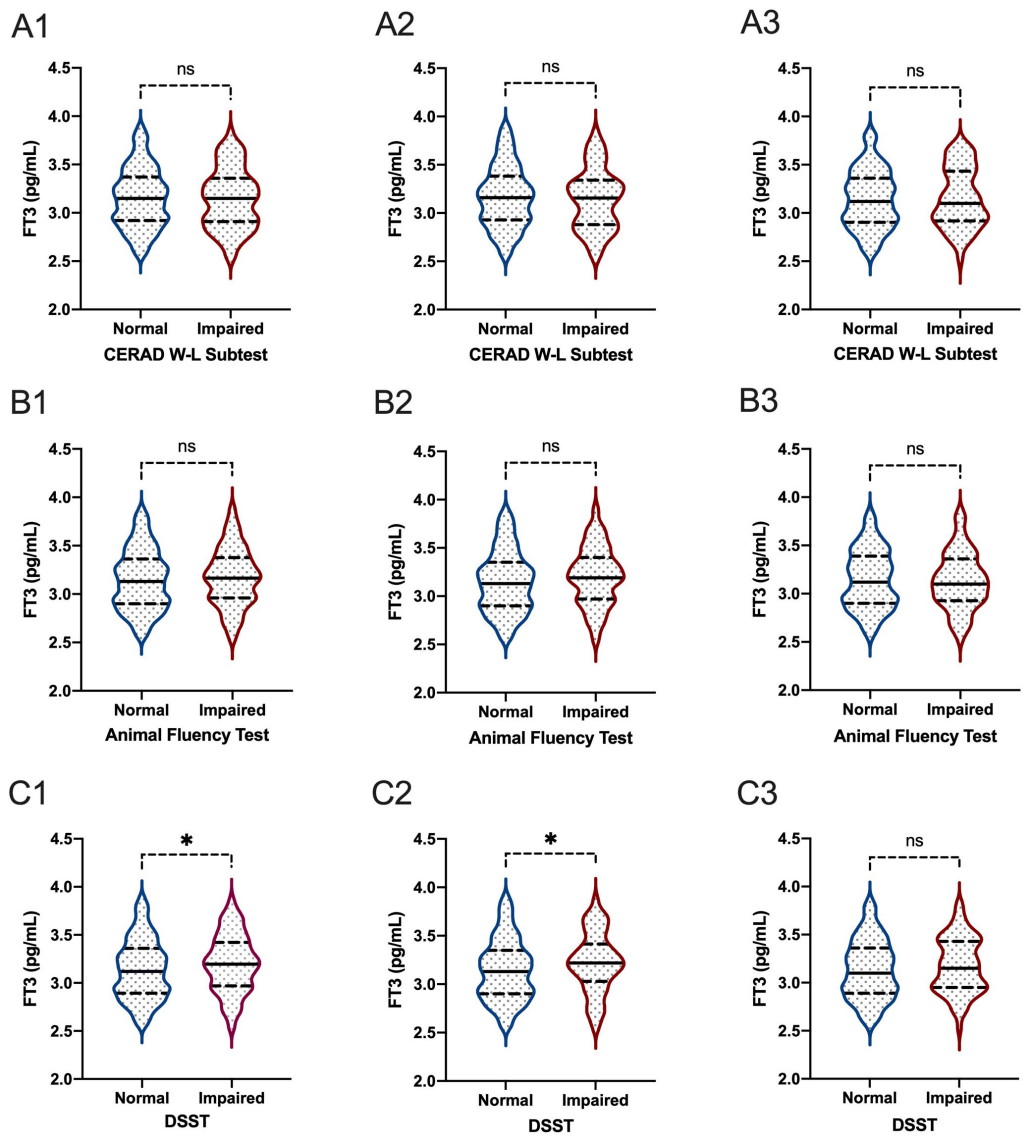
The comparisons of FT3 between participants with and without impaired cognitive functioning. **(A1-3)**: for CERAD W-L Subtest; **(B1-3)** for Animal Fluency Test; **(C1-3)** for DSST; **(A1, B1, C1)** for overall participants; **(A2, B2, C2)** for male participants; **(A3, B3, C3)** for female participants. *P < 0.05. ns: not significant.

**Figure 6:**
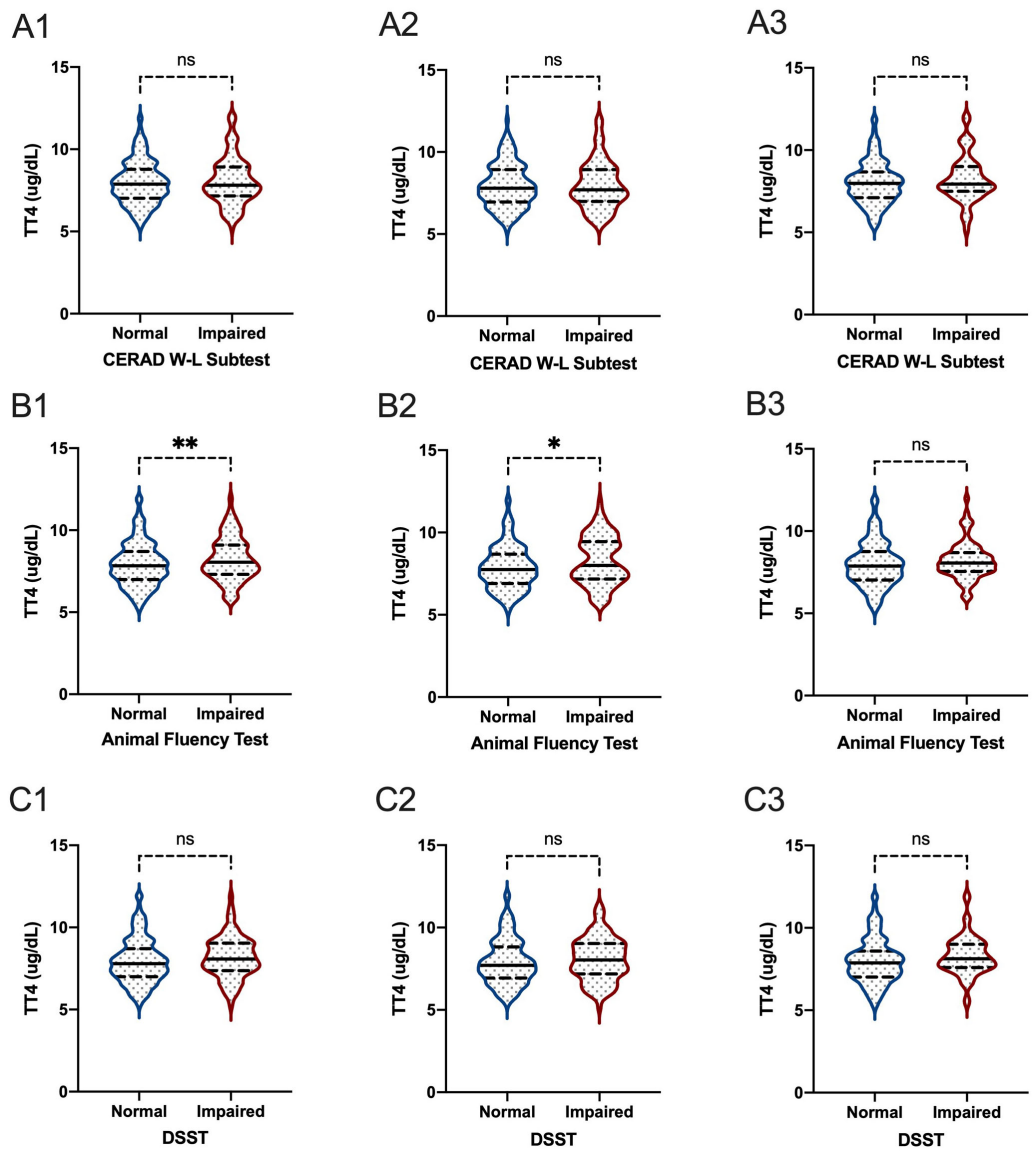
The comparisons of TT4 between participants with and without impaired cognitive functioning. **(A1-3)** for CERAD W-L Subtest; **(B1-3)** for Animal Fluency Test; **(C1-3)** for DSST; **(A1, B1, C1)** for overall participants; **(A2, B2, C2)** for male participants; **(A3, B3, C3)** for female participants. *P < 0.05; **P < 0.01. ns: not significant.

When comparing thyroid function, including FT3, FT4, TSH, TT3, and TT4, between participants with normal cognitive functioning and participants with impaired cognitive functioning, we found that there were significant differences between participants with and without impaired cognitive functioning for FT3 level in all participants based on the DSST score (P=0.020) (**Figures 5**, **6**). In the Animal Fluency Test, participants with impaired cognitive functioning had significantly higher TT4 levels than those in the normal cognitive functioning group (P=0.006). After stratifying by sex, significant differences were observed in male participants in the Animal Fluency Test and DSST (P=0.031 and P=0.011, respectively). Interestingly, there was no association in female participants (all P>0.05) (**Supplementary File 1**).

## 4 Discussion

To the best of our knowledge, this is the first population-based cross-sectional study to investigate the association between thyroid hormones and the three dimensions of cognitive functioning in older euthyroid adults using the NHANES database. Evidence suggested that changes in THs can have intensive effects on the modulation of human behavior, especially as to neurocognitive and neuropsychiatric aspects (28). Venero et al. found that a 10-fold reduction in the affinity for THs was observed when thyroid hormone receptor α1 (TRα1) gene was mutated, which resulted in decreased exploratory capacities, locomotor dysfunctions and memory deficits (29). This echoed to another study where the adults mouse model with imbalanced THs has been shown to yield spatial memory deficits (30). Furthermore, Hochbaum et al., revealed that a cortical-neuron-induced T3-sensitive transcriptional program in male mice altered cortical circuits and increased exploration and risk taking in multiple contexts (31). As such, these neurocognitive and neuropsychiatric disorders may be compensated by pharmacological administration of T3. Thus, our study lays the foundation for elucidation of the intricated association the thyroid hormones and cognitive functioning.

The results revealed a significant inverse relationship between FT3 levels within the normal range and the two dimensions of cognitive functioning (i.e., the Animal Fluency Test and DSST) after adjustment for potential covariates. This suggested that higher FT3 levels within the normal range were associated with poorer cognitive performance, which was in line with the study conducted by Grigorova and Sherwin wherein higher FT3 levels were positively associated with longer completion time (poorer performance) on the Trail Making Test—Part A (P=0.006) and Part B (P=0.032) and the Tower of London Test (P=0.002) (8). Importantly, a mono-center study found that higher levels of serum FT3 were associated with lower risk of conversion to AD (HR=0.54, 95% CI: 0.32-0.92 per 1 pmol/L increase) (9). However, due to its small scale (302 participants, of whom only 55 were diagnosed with AD), the results of the aforementioned study should be interpreted cautiously. Apart from the different sample sizes, the criteria for determining cognitive impairment or decline accounted for inconsistent findings, in part. Generally speaking, FT3 might be a future predictive hormone to assess cognitive decline or impairment through the thyroid function–cognition loop.

The subgroup analysis stratified by sex suggested that the inverse relationship between FT3 and cognitive functioning, as measured by the Animal Fluency Test, remained significant in both men and women. Interestingly, for DSST, a negative association was observed only in female participants. Similarly, another study, with a total of 122 euthyroid women, demonstrated that higher FT3 levels within the reference range were correlated with snail-like performance and more errors in executive functioning tests (8). Additionally, Volpato et al. reported an association between T4 levels within the normal range and the risk of cognitive decline (32). In contrast, Prinz et al. found that there was a positive correlation between TT4 levels within the normal range and cognitive performance among healthy men with a mean age of 72 years (33). Furthermore, a population-based Heinz Nixdorf Recall study showed a strong association between high-normal TSH concentration and MCI in women rather than men (34). The discrepancies in findings may be explained by heterogeneity between studies, including methodological differences (e.g., the various sample sizes and study designs).

It is worth noting that no association was found between TSH and cognitive functioning in our study in either weighted multivariable linear regression or binary logistic regression analyses, which was in line with previous studies (35, 36). However, Beydoun et al. reported that TSH levels were associated with poor performance on various neuropsychological tests in individuals aged between 20 and 59 years, but better performance in older adults from 60 to 90 years using the NHANES III sample (37). Likewise, Moon et al. found that every 1 mIU/L decrease in serum TSH was associated with an approximately 1.7 times increased risk of the progression of cognitive impairment over 5 years (10). Although it is unclear why TSH was not associated with three tests of cognitive functioning, it is likely that the network of cognition related to TSH is complicated and dependent on the activation of multiple neural pathways and the distribution of corresponding receptors in the brain. Further research should be conducted to reveal the mechanism for better understanding the relationship between these two variables.

The results showed that there were significant differences between participants with and without impaired cognitive functioning for serum FT3 levels in overall participants based on DSST score (P=0.020). A cross-sectional and multi-institutional joint study reported that FT3 levels in both males and females with and without cognitive impairment decreased with age (P<0.0001) (38). The average FT3 values for both males and females without cognitive impairment were significantly higher than those with cognitive impairment (38). In our study, significant differences were observed for the Animal Fluency Test and DSST in males rather than females. However, this does not imply that interventions or management are rarely required for females.

As indicated in our study, THs were associated with the two dimensions of cognitive functioning (i.e., the Animal Fluency Test and DSST) rather than the global cognition. The Animal Fluency Test is a compartment of executive function to examine verbal category fluency. The study conducted by Choi and his colleagues unveiled that FT4 was inversely correlated with attention and visuospatial and executive dysfunctions, while an association of FT4 with executive dysfunction was only shown in multivariate analyses (39). This finding was expounded by a high FT4 level associated with smaller hippocampal volumes on MRI scans of non-demented elderly (40). For DSST, a performance module aims to examine the participant’s processing speed, visual scanning, sustained attention and working memory. Schraml et al. found that the overt hypothyroidism induced iatrogenically was associated with cognitive impairments restricted to memory, specifically working memory, compared to a control group (41). Smith et al. found that transient profound hypothyroidism was marked by reversible depression, decreased fine motor performance, slowed reaction times and reduced processing speed (42). In our study, we also observed the inverse relationship between FT3 and DSST score. For working memory indices of DSST, this finding can be explicated by the study where the hippocampus is incredibly sensitive to T3, and relative excess may manifest in aggravated episodic memory dysfunctions (43). In term of global cognitive function, the study of individual participant data analysis 18 cohorts failed to find the association between subclinical thyroid dysfunction and global cognitive function (13). Therefore, we need target to the significant effects of THs on specific cognitive domains, such as visuospatial function and working memory, rather than global cognitive function. Yet the improvements of specific cognitive performance will contribute to enhancing global cognitive function.

Our study has several strengths. For example, the data were retrieved from the NHANES database of the U.S. National Center for Health Statistics, ensuring the representativeness and generalizability of our study. In addition, this is the first study to focus on the U.S. population to determine the relationship between thyroid hormones and cognitive functioning in older euthyroid adults. However, our study had some limitations. First and foremost, the causal relationship between thyroid hormones and cognitive functioning was not examined due to the cross-sectional study design of the NHANES survey. Second, many other potential factors, except for the covariates used in our study, could have influenced the relationships between independent variables. Finally, cognitive functioning was assessed using three specific tests available in the NHANES, which may not comprehensively cover all domains of cognitive functioning.

## 5 Conclusion

In summary, this study demonstrated a significant inverse relationship between FT3 levels within the normal range and two dimensions of cognitive functioning, the Animal Fluency Test and DSST, after adjustment for potential covariates. Importantly, a significant negative association remained for females and DSST in the subgroup analysis stratified by sex. Binary logistic regression showed that the significantly increased aORs (95% CI) between the risk of impaired cognitive functioning and FT3 across Q3 and Q4 compared with Q1 were 2.025 (1.092, 3.753) and 2.365 (1.261, 4.433), respectively, for DSST in overall participants. Furthermore, there were significant differences between participants with and without impaired cognitive functioning for serum FT3 levels in overall participants based on DSST score (P=0.020). Future longitudinal cohort studies should be conducted to determine the causal relationship between thyroid hormone levels and cognitive functioning.

## Data Availability

The raw data supporting the conclusions of this article will be made available by the authors, without undue reservation.
