# Supplementary material for: Association between thyroid hormones and cognitive functioning in euthyroid elderly adults: a cross-sectional preliminary study from the NHANES 2011–2012 survey
Source: Front Endocrinol (Lausanne). 2024 Nov 8;15:1476086. doi: 10.3389/fendo.2024.1476086 (PMC11581855; doi:10.3389/fendo.2024.1476086)
Supplement: Supplementary file 1 [file DataSheet1.pdf]

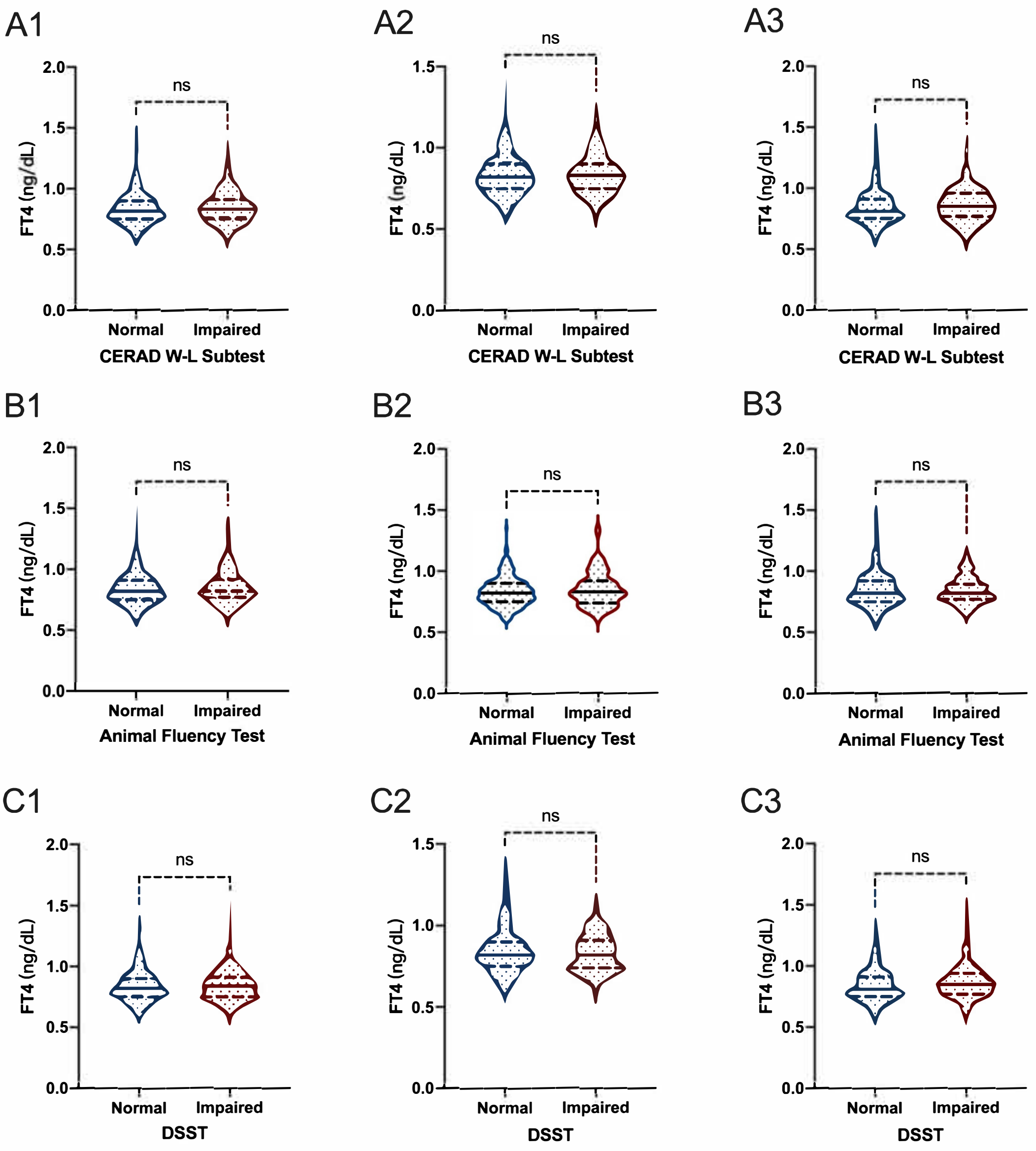

Supp1ementary Figure 1. The comparisons of FT4 between participants with and without impaired cognitive functioning. A1-3: for CERAD W-L Subtest; B1-3: for Animal Fluency Test; C1-3: for DSST; A1, B1, C1: for overall participants; A2, B2, C2: for male participants; A3, B3, C3: for female participants.

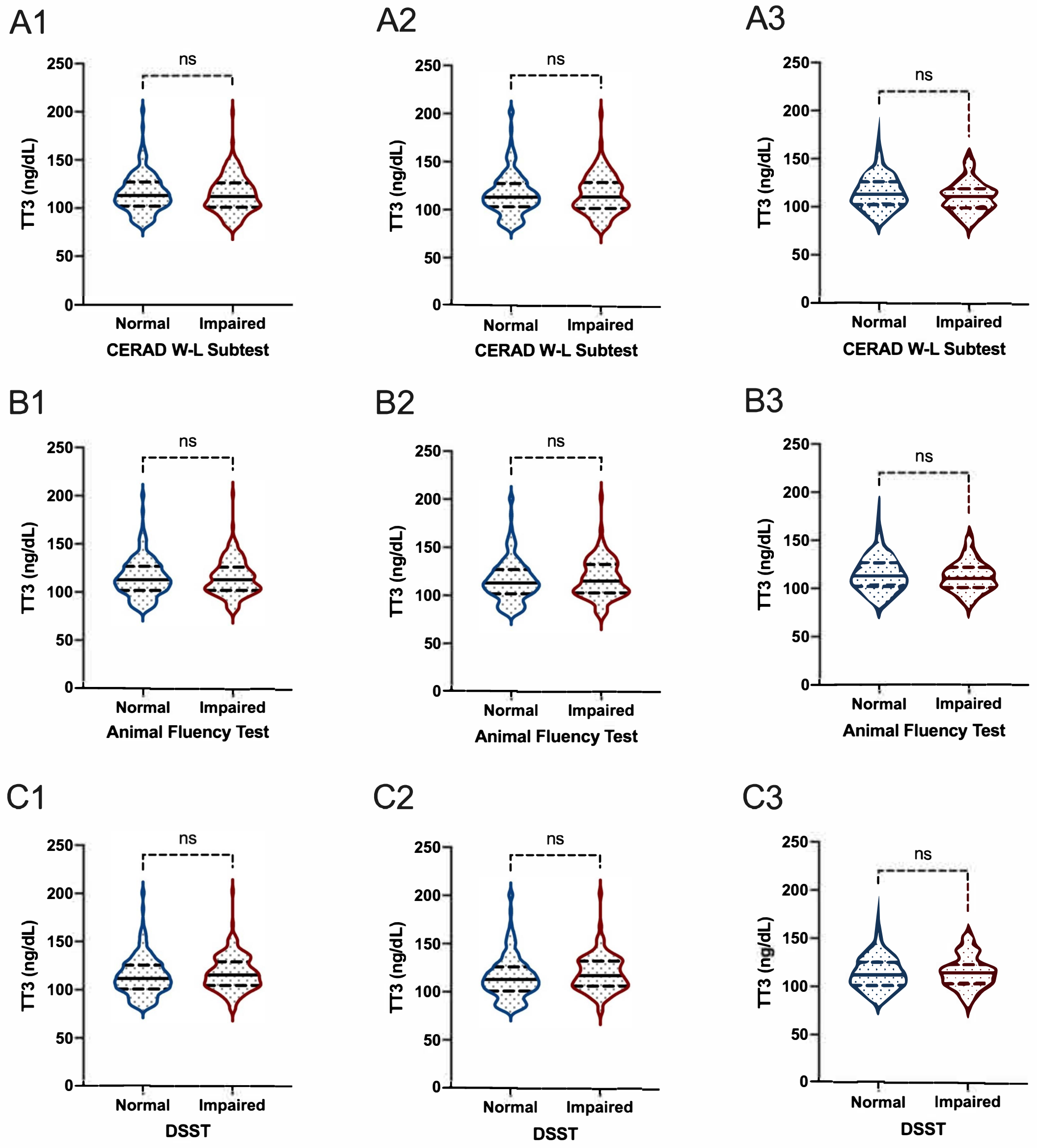

Supp1ementary Figure 2. The comparisons of TT3 between participants with and without impaired cognitive functioning. A1-3: for CERAD W-L Subtest; B1-3: for Animal Fluency Test; C1-3: for DSST; A1, B1, C1: for overall participants; A2, B2, C2: for male participants; A3, B3, C3: for female participants.

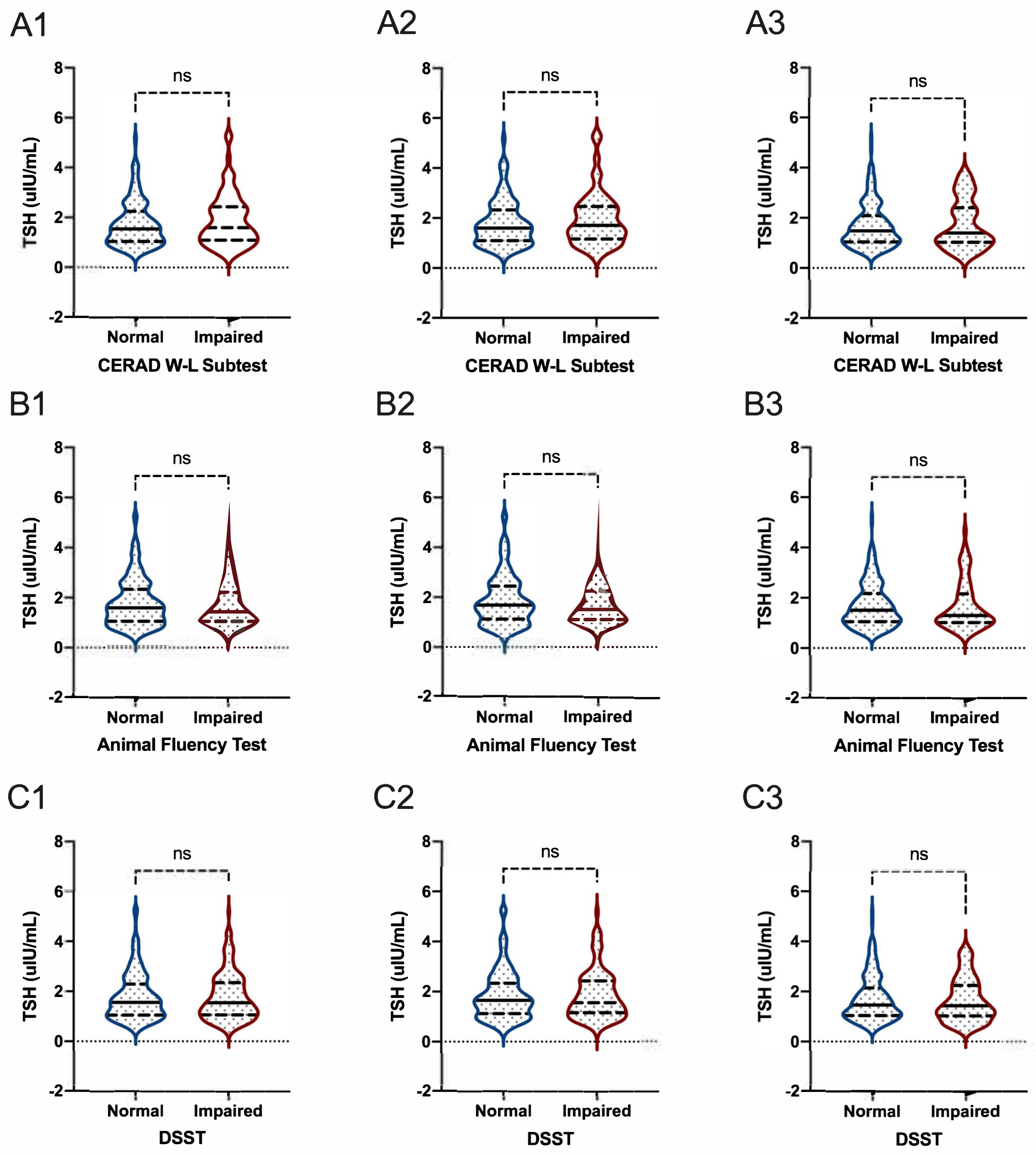

Supp1ementary Figure 3. The comparisons of TSH between participants with and without impaired cognitive functioning. A1-3: for CERAD W-L Subtest; B1-3: for Animal Fluency Test; C1-3: for DSST; A1, B1, C1: for overall participants; A2, B2, C2: for male participants; A3, B3, C3: for female participants.
